# ^18^F-NaF uptake on vascular PET imaging in symptomatic versus asymptomatic atherosclerotic disease: A meta-analysis

**DOI:** 10.1177/1358863X241287692

**Published:** 2024-10-16

**Authors:** Shiv Bhakta, Mohammed M Chowdhury, Jason M Tarkin, James HF Rudd, Elizabeth A Warburton, Nicholas R Evans

**Affiliations:** 1 Department of Clinical Neurosciences, University of Cambridge, Cambridge, UK; 2 Department of Vascular Surgery, University of Cambridge, Cambridge, UK; 3 Division of Cardiovascular Medicine, University of Cambridge, Cambridge, UK

**Keywords:** atherosclerosis, microcalcification, positron emission tomography (PET), vascular imaging/diagnostics, risk stratification

## Abstract

**Introduction:**

^18^F-sodium fluoride (NaF) positron-emission tomography (PET) is increasingly being used to measure microcalcification in atherosclerotic disease in vivo. Correlations have been drawn between sodium fluoride uptake and the presence of high-risk plaque features, as well as its association with clinical atherosclerotic sequelae. The aim of this study was to perform a meta-analysis of NaF uptake on PET imaging and its relation to symptomatic and asymptomatic disease.

**Methods:**

A systematic review was performed according to PRISMA guidelines, via searching the Ovid MEDLINE, Ovid Embase, Cochrane Library, PubMed, Scopus, and Web of Science Core Collection databases up to May 2024. The search strategy included the terms ‘NaF’, ‘PET’, and ‘plaque’, and all studies with data regarding the degree of microcalcification, as measured by ^18^F-NaF uptake in symptomatic and asymptomatic atherosclerotic plaques, were included. Analysis involved calculating mean differences between uptake values and comparison using a random-effects model.

**Results:**

A total of 16 articles, involving 423 participants, were included in the meta-analysis (10 carotid artery studies, five coronary artery studies, and one in peripheral vascular disease). Comparing ^18^F-NaF uptake in symptomatic versus asymptomatic atherosclerotic plaques, a mean difference of 0.43 (95% CI 0.29 to 0.57; *p* < 0.0001, *I*^2^ = 65%) was noted in studies comparing symptomatic and asymptomatic plaques in the same participant, with a significant difference in effect based on arterial territory studied (χ^2^ = 12.68, *p* = 0.0018). In studies of participants with and without symptomatic disease, there was no significant difference between symptomatic and asymptomatic plaques (mean difference 0.27, 95% CI −0.26 to 0.80, *p* = 0.28, *I*^2^ = 85%).

**Conclusions:**

PET imaging using ^18^F-NaF can detect differences in microcalcification between symptomatic and asymptomatic atherosclerotic plaques within, but not between, individuals, and thus, is a marker of symptomatic disease. The standardization of ^18^F-NaF PET imaging protocols, and its future use as a risk stratification tool or outcome measure, requires further study. **(PROSPERO Registration ID: CRD42023451363)**

## Introduction

Atherosclerosis is a systemic chronic arterial disease,^1^ involving the accumulation of lipids and inflammatory cells^2^ to form foci of disease, termed ‘plaques’, at the vessel wall. It is the cause of over one-third of all deaths,^3^ through resulting diseases such as myocardial infarction, ischemic stroke, and critical limb-threatening ischemia. In some patients, minor or less severe clinical symptoms may be a marker of higher risk for progressing to more severe clinical disease, such as stable angina preceding myocardial infarction,^4,5^ or transient ischemic attack, conferring a higher risk of ischemic stroke in the short term.^6,7^

Plaques may have heterogenous appearances,^8^ with certain plaque features indicating a higher risk of rupture and subsequent clinical sequelae.^8,9^ These high-risk features include the presence of a lipid-rich necrotic core,^10^ intraplaque hemorrhage,^11^ a thin or ruptured fibrous cap,^12^ and the presence of microcalcification.^13^ The mechanisms determining the transition from a low-risk (‘stable’) to a high-risk (‘unstable’) plaque and vice versa are incompletely understood,^14,15^ but microcalcification has been recognized as a potential cause for acute plaque rupture through mechanical destabilization of the fibrous cap of the plaque,^16^ as well as causing an increased inflammatory response within the plaque,^17^ leading to enzymatic destabilization of the plaque.^18^ Microcalcifications are calcium deposits of < 50 μm in diameter, which is below the spatial resolution of commonly used clinical vascular imaging techniques,^19^ such as computed tomography (CT) or magnetic resonance imaging (MRI).

Positron emission tomography (PET) is a nuclear imaging technique used in vascular imaging due to its high sensitivity to detect low concentrations of radiolabeled ligands (termed ‘tracers’), which can be directed to detect the presence of a specific target or process.^20^ Sodium fluoride (NaF), labeled with fluorine-18, has been validated as a tracer for the identification of microcalcification in vascular imaging.^21^

NaF adsorbs to the surface of hydroxyapatite within the body,^22^ with hydroxyapatite being the most common calcium-containing crystal structure in atherosclerotic plaques in vivo.^23,24^ Fluorine exchanges with hydroxyl groups on the surface of these crystals, with substantially less tracer uptake deeper within the crystal structure of the molecule.^22^ Calcium deposits with an increased surface area therefore have increased fluoride ion uptake over the uptake durations used in clinical PET scanning.^22,25^ Using NaF, this process of hydroxyl-ion substitution can be used to detect microcalcifications via PET imaging. Owing to their high surface area to volume ratio, microcalcifications will demonstrate higher uptake on PET compared to areas with no microcalcification, or those with larger deposits of calcium (‘macrocalcification’).^26–28^ NaF-PET can be preferred for vascular imaging compared to other PET imaging techniques for visualizing high-risk plaques due to issues such as ‘spill-over artifact’ when using fluorodeoxyglucose (FDG)-PET in cardiac imaging.

There is an increasing body of literature demonstrating the use of NaF-PET for vascular imaging in atherosclerosis,^29^ and specifically, in symptomatic disease. This meta-analysis focuses on the role of NaF-PET imaging to differentiate between symptomatic versus asymptomatic atherosclerotic disease.

## Methods

### Protocol, search strategy, and selection criteria

Details for the protocol were prospectively registered on PROSPERO (ID: CRD42023451363).^30^ The selection process and reporting items were based on the preferred reporting items for systematic reviews and meta-analysis (PRISMA) flow diagram and checklist.^31^ The primary outcome was to determine differences in NaF tracer uptake between symptomatic and asymptomatic atherosclerotic plaques. A search strategy was formulated using Embase and MEDLINE via Ovid, the Cochrane Library, PubMed, Scopus, and Web of Science Core Collection Databases (Supplementary Figures S1a–e). Additionally, a manual search was performed to identify relevant records through reference searches. Duplicate records were removed, and the retrieved records were checked for inclusion and exclusion criteria.

Studies were eligible for inclusion if they investigated the association between NaF uptake and symptomatic and asymptomatic atherosclerotic plaques, where symptomatic plaques were those that were associated with a recent clinical vascular event, including, but not limited to, stroke, transient ischemic attack, or myocardial infarction, diagnosed by recognized clinical or imaging criteria, whereas asymptomatic plaques were those not associated with a recent clinical vascular event. Studies were excluded if animal or preclinical data were used, nonatherosclerotic or nonsymptomatic atherosclerotic disease was investigated, or if the study did not provide details regarding the type or location of symptomatic disease. In addition, studies not using NaF-PET were excluded, along with editorial or review articles and case reports. Abstracts were eligible for inclusion in the systematic review and meta-analysis to ensure the completeness of the data obtained, and to ensure the most recent research was included in this emerging field.

### Data selection, extraction, and risk of bias assessment

Data were extracted by one study investigator (SB) and checked by another researcher (NRE), from the included records, using a standardized electronic data collection form. Discrepancies were resolved by re-extraction or by third-party adjudication (EAW) as required. Retrieved characteristics from the studies included, but were not limited to, the number of participants, participant population, targeted vascular territory, time from symptomatic event to imaging, dose of NaF injected, uptake time, imaging protocols, primary endpoint measures for PET/CT, and main findings, which were tabulated as per published guidance.^32^ The Risk of Bias in Observational Studies of Exposures (ROBINS-E) tool^33^ was used to assess the risk of bias of the studies included in the analysis.

### Statistical analysis

The mean and associated SD, or the median and the associated IQR of the measurement of NaF signal were extracted from the included studies. Extracted median and IQR data were converted into mean and SD data to calculate a unified outcome.^34^ In studies where multiple measures of NaF signal were reported, the value corresponding to the tissue-to-background ratio (TBR) related to the maximum standardized uptake value (SUV) (TBR_max_) was taken. The absolute difference between the populations when NaF signal is measured using SUV versus TBR is minor, given the low blood pool activity of NaF following an appropriate uptake time.^19^

Studies were classified by their included participants – those with only symptomatic participants, or those also including an asymptomatic control population. Given that considerable between-study heterogeneity was anticipated, random-effects models were used for meta-analysis. We used Knapp–Hartung adjustments^35^ to calculate the confidence interval around the pooled effect. The restricted maximum likelihood estimator^36^ was used to calculate the between-study variance. *I*^2^ statistics were also calculated to determine the variability in effect estimate due to between-study heterogeneity. Meta-analyses were performed where there were two or more studies using the same type of included population. Pooled outcomes from the included studies were reported as mean differences.

Funnel plot asymmetry was assessed visually and using Egger’s test,^37^ where 10 or more studies were included in the meta-analysis to identify any small study effects. Two-tailed tests were used, and a *p*-value of 0.05 was taken as the limit of statistical significance. Statistical analyses were performed using the *meta*^38^ package, using R Statistical Software (v4.3.1; R Foundation for Statistical Computing, 2023), with mean and SD values estimated from extracted median and IQR data by the Method for Unknown Non-Normal Distributions.^39^

Subgroup analyses were performed between the different arterial territories assessed in the included studies to generate mean difference measurements for symptomatic atherosclerotic plaques compared with asymptomatic plaques, and to determine any statistical differences in NaF uptake between different arterial territories.

## Results

### Included studies

**Figure 1.**
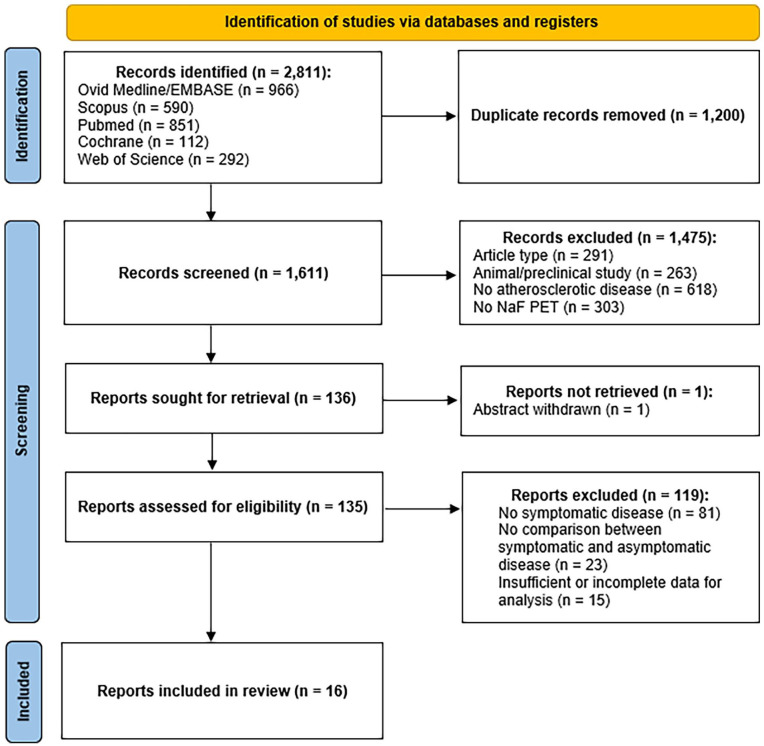
PRISMA diagram of systematic review search synthesis.

A total of 2811 titles were initially identified from the search (Figure 1).^40^ Manual de-duplication of results was performed, and the remaining 1611 records underwent manual screening of the titles and abstracts.^41^ Of those, 1475 were excluded as not meeting the inclusion criteria (618 not related to atherosclerotic disease, 303 not related to NaF-PET imaging, 291 being of the incorrect article type, and 263 reporting only animal or preclinical data). A total of 136 articles were therefore sought for retrieval. One article was not available for analysis due to the abstract being withdrawn, and therefore 135 articles were assessed for eligibility through full-text review. Of these, 119 were excluded (81 due to not reporting outcomes related to symptomatic atherosclerotic disease, 23 due to no documented comparison between symptomatic and asymptomatic disease, and 15 due to reporting data or outcome measures insufficient for meta-analysis). The remaining 16 articles were included in the meta-analysis and form the study population analyzed. A summary of study details is shown in Supplementary Tables S1 and S2, dichotomized based on the comparator of symptomatic disease used in each study.

### Comparative intra-individual ^18^F-NaF tracer uptake between symptomatic and asymptomatic atherosclerotic disease

Twelve studies, including 312 participants, reported data in participants comparing NaF uptake in the symptomatic atherosclerotic plaque to asymptomatic plaques within the same participant’s vascular territory being observed, including data for 312 symptomatic and 377 asymptomatic plaques (Supplementary Table S1).

**Figure 2.**
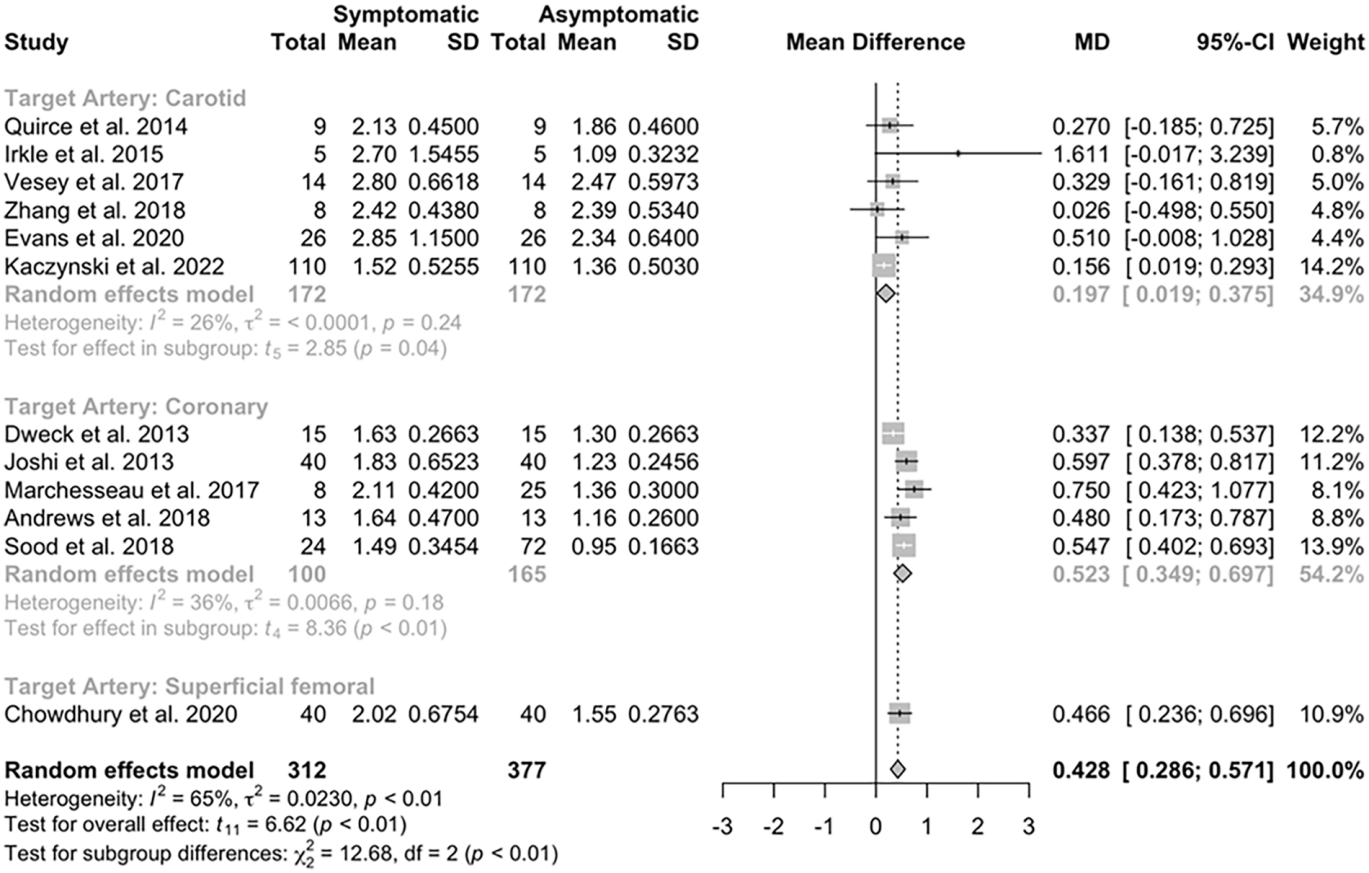
Forest plot of included studies^25,41,52,69–77^ summarizing data comparing symptomatic and asymptomatic atherosclerotic disease within individuals. MD, mean difference.

Pooled comparisons of these studies demonstrated a significantly higher uptake in symptomatic lesions compared to asymptomatic plaques (mean difference 0.43, 95% CI 0.29 to 0.57, *p* < 0.0001; Figure 2). A funnel plot (Supplemental Figure S2) assessing standard error about the mean difference in the included studies did not appear asymmetric visually, and the result of Egger’s test was not statistically significant (*p* = 0.33). Subgroup analysis to compare results from different arterial territories showed a significant difference in this relationship based on the site of the symptomatic plaque (χ^2^ = 12.68, *p* = 0.0018). Significant heterogeneity was noted between the studies (τ^2^ = 0.023, *I*^2^ = 65%, *p* = 0.00097).

A sensitivity analysis was performed, including those studies found to be at low risk of bias other than due to residual confounding or where there were some concerns about risk of bias only in one domain, due to percutaneous coronary intervention being indicated clinically in participants with myocardial infarction (Supplementary Figure S3). This analysis, which included three carotid artery studies, one coronary artery study, and one study investigating the superficial femoral arteries, showed similar findings to the parent analysis (mean difference 0.39, 95% CI 0.11 to 0.68, *p* = 0.019; Supplementary Figure S4). This sensitivity analysis excluded data obtained from nonpeer-reviewed articles^42–44^ as these were deemed to be at high risk of bias.

### Comparative 18F-NaF tracer uptake in symptomatic and asymptomatic atherosclerotic plaques where a healthy control population was included

Eight studies, including 212 participants, reported data in participants comparing NaF uptake in the symptomatic atherosclerotic plaque compared to asymptomatic plaques, pooling data from asymptomatic disease within the same symptomatic individual and plaques from asymptomatic control participants, including data for 189 symptomatic and 189 asymptomatic plaques (Supplementary Table S2). Six studies assessed atherosclerotic disease within the carotid arteries, with the remaining two studies assessing disease within the coronary arteries. The risk of bias assessment for these studies is shown in Supplementary Figure S5.

**Figure 3.**
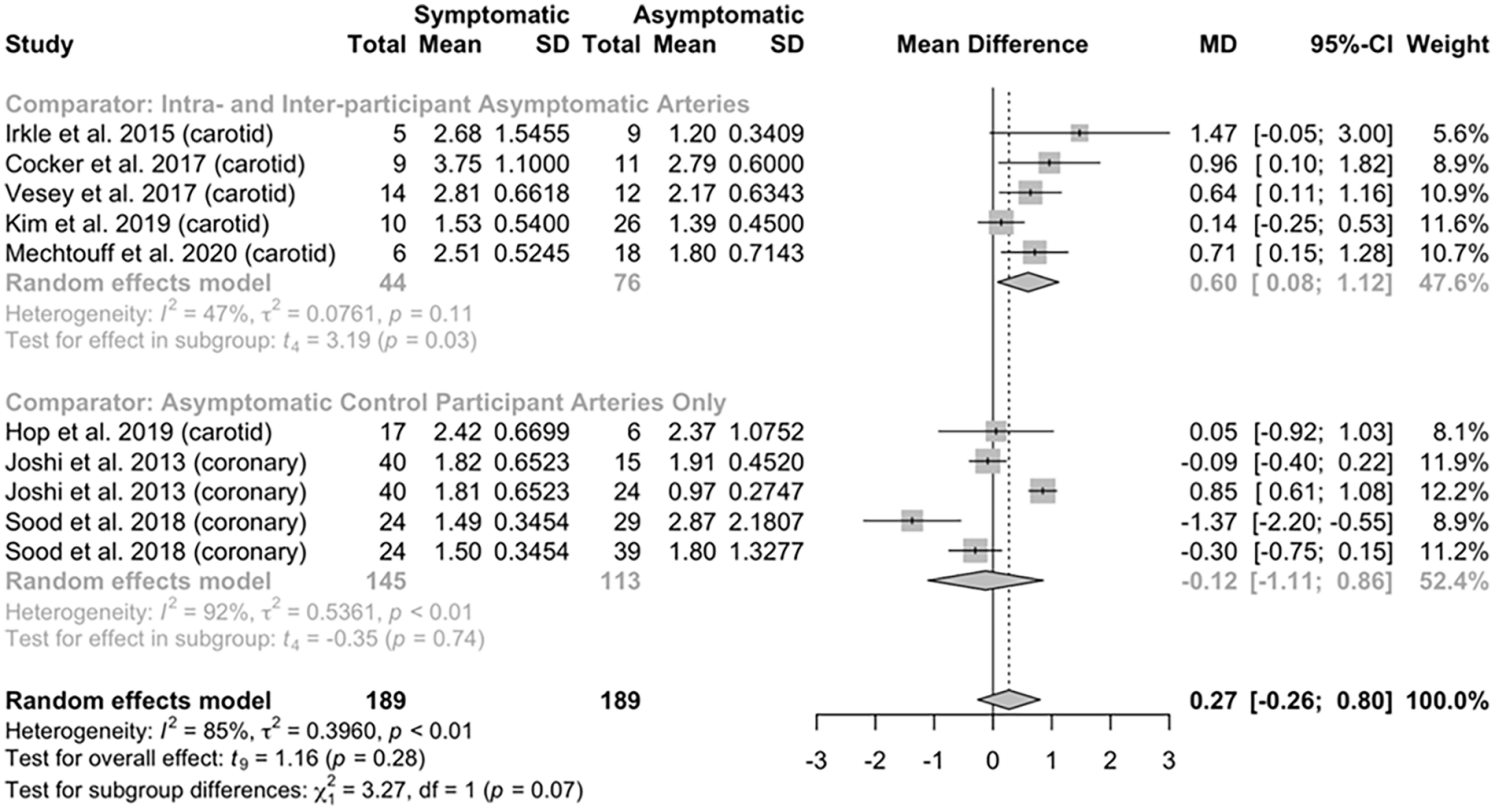
Forest plot of included studies^25,70,72,75,78–81^ summarizing data comparing symptomatic and asymptomatic atherosclerotic disease between individuals. MD, mean difference.

Analysis of the pooled data from these studies demonstrated no significant overall difference in NaF uptake in symptomatic atherosclerotic lesions compared to asymptomatic lesions, present in either the same individuals or in healthy controls (mean difference 0.27, 95% CI −0.26 to 0.80, *p* = 0.28; Figure 3). Subgroup analysis was performed to investigate differences between studies assessing NaF uptake in asymptomatic control participants only, or in asymptomatic control participants and asymptomatic plaques within a symptomatic participant. Studies investigating asymptomatic plaques within and between participants were solely performed in the carotid arteries, and in this group of five studies, symptomatic arteries showed a higher NaF uptake (mean difference 0.60, 95% CI 0.08 to 1.12, *p* = 0.03), whereas those studies including asymptomatic participants only as comparators, without assessing asymptomatic plaques within the symptomatic individual (two coronary artery studies and one carotid artery study), showed no significant difference in NaF uptake (mean difference −0.12, 95% CI −1.11 to 0.86, *p* = 0.74). There was significant heterogeneity between studies included in this analysis (τ^2^ = 0.40, *I*^2^ = 85%, *p* < 0.0001).

## Discussion

These results demonstrate the utility of NaF-PET, combined with CT or MRI, to differentiate between symptomatic and asymptomatic atherosclerotic disease within individuals in a number of different vascular territories. A mean difference of 0.43 units in the meta-analysis of studies included in Figure 2 shows the sensitivity and reproducibility^45^ of PET as an imaging modality for the investigation of atherosclerosis, and the detection of differing levels of microcalcification between plaques in at-risk individuals using commonly used imaging protocols and analysis methods. In the context of potential clinical ramifications, in a small study, a one-unit increase in TBR_max_ in the carotid arteries has been associated with an up to 31% increased risk of recurrent ipsilateral stroke.^46^ Studies have also demonstrated a correlation between NaF uptake and high-risk morphological features on MRI, such as the presence of a lipid-rich necrotic core, or intraplaque hemorrhage.^47^ In addition, there is emerging evidence of a prognostic link between NaF signal on PET and the risk of recurrent disease,^48^ where coronary NaF imaging had the ability to predict myocardial infarction and cardiovascular death in symptomatic patients. NaF-PET imaging also has been shown to predict future symptomatic coronary disease in high-risk individuals.^49^

As noted in Supplementary Tables S1 and S2 and Figures 2 and 3, there is significant clinical and methodological heterogeneity between studies. We utilized random-effects models in this analysis given these noted differences had the potential to affect the outcome data, and specifically the magnitude of difference between the two groups. These differences are in part due to the clinical diversity of the populations included in the study, including different research sites, with associated differences in the prevalence and diagnosis of the relevant atherosclerotic disease, and participants undergoing clinical interventions prior to imaging, such as percutaneous coronary intervention in some participants with myocardial infarction, with the effects on NaF uptake not well described in the literature. There is also significant heterogeneity in the results due to the methodological variation of the included studies, including a range of different doses of NaF radiotracer used, along with a nonstandardized uptake time, blood pool measurement for TBR calculations, and outcome measurement, including how and where maximum uptake was calculated. Irkle et al.^25^ demonstrated an optimum uptake time of around 60 minutes, based on in vitro and in vivo data, and a 60-minute uptake time was the modal NaF uptake time in the included studies. This heterogeneity may introduce confounding factors, but the study participants seem to represent the range of patients seen in clinical practice. In addition, the results appear in keeping with the larger body of literature describing NaF-PET in atherosclerosis due to its associations with high-risk plaque histology^50,51^ and imaging appearance,^52,53^ and its links with new^49,54^ or symptomatic disease.^46,48^

There was also significant heterogeneity in the included studies between the time of symptom onset and imaging being performed (Supplementary Tables S1 and S2).^82,83^ Insufficient data are available concerning the temporal changes in microcalcification and NaF uptake following an acute atherosclerotic event, and further investigation of the optimum time for imaging microcalcification in relation to symptomatic disease, and further data on the change in microcalcification with time following symptomatic disease, could further standardize imaging protocols and improve the robustness of outcomes measured using NaF-PET.

NaF-PET lacks a consensus on best practice, in contrast to vascular FDG-PET imaging, following the 2016 position paper from the European Association of Nuclear Medicine.^55^ Having a standardized methodology for performing and analyzing vascular NaF imaging would allow increased comparability and reproducibility between studies. Aspects for standardization would include the administered radiotracer dose, duration between dose administration and imaging, and between clinical event and imaging, along with imaging protocols and analysis, such as how imaging should be performed in order to best remove motion artifact, as well as how to measure and describe uptake levels, which could include plaque-based or anatomical uptake, SUV versus TBR (or alternatives^56^), and whether the TBR_max_ should be reported, or some other measure, such as the ‘most diseased segment’, being the mean of the TBR_max_ over the region of interest (ROI) with the highest reading and the TBR_max_ of the two adjacent ROIs.^55,57^ In addition, in comparisons involving intra-participant imaging data, description and analysis in a paired manner may be an interesting avenue for future research in this area, as this could provide information as to the differences in uptake between symptomatic and asymptomatic plaques, removing some confounding due to underlying genetic and physiological differences between individuals.

Of note, in the subgroup analysis, there appeared to be a reduced mean difference between symptomatic and asymptomatic disease within the same individual in the carotid artery studies. Though this may be a result of the clinical and methodological heterogeneity discussed above, an underlying pathophysiological difference in how carotid artery plaques behave in vivo compared to other sites of atherosclerosis is also biologically plausible. Some studies have suggested relationships between vessel wall shear stress and NaF uptake,^58^ the effects of vessel geometry on shear stress,^59,60^ and differences in shear stress in different arterial territories,^61^ which theoretically could lead to increased microcalcification in carotid atherosclerotic plaques, leading to the smaller difference between symptomatic and nonculprit carotid plaques within the same individual, as seen in the above analysis. Future research investigating NaF uptake in different arterial territories simultaneously would help answer this question of differential uptake, and the associated clinical implications; for example, relative risks of future disease within each territory, and whether different clinical management may be recommended based on the site of highest uptake.

In the analysis of culprit vessels compared to nonculprit vessels, including those in asymptomatic control participants, no overall mean difference between culprit and nonculprit plaques was demonstrated. This follows from the above discussion, as NaF-PET imaging in atherosclerosis has been used to identify symptomatic disease, as well as high-risk disease in asymptomatic individuals, suggesting NaF-PET may not be able to distinguish between presymptomatic (but high-risk) and symptomatic plaques. Therefore, rather than providing a threshold value, over which any plaque in any person may be deemed ‘high-risk’, this analysis demonstrates that comparison of plaques within individuals rather than between individuals is more useful to identify high-risk or symptomatic plaque. This would also concur with the current understanding of the underlying physiology around microcalcification, where there is a higher risk of plaque rupture with increasing microcalcification due to induced changes and responses to wall shear stress,^62,63^ rather than a threshold level of microcalcification below which the plaque is stable, and above which confers risk of disease.

In atherosclerosis, PET has been demonstrated to have a high sensitivity for the target pathophysiology and can be combined with other imaging modalities to provide additional utility, such as in combination with CT and CT angiography to assess for stenotic disease^64^ and calcium scores in the coronary vasculature,^65^ or with MRI to identify high-risk appearances as discussed above.^66^ In vascular imaging, NaF may be superior to FDG as it may have a superior ability to discriminate between symptomatic and asymptomatic disease in high-risk individuals.^67^ In addition, NaF does not require fasting prior to the uptake period, and can be used with a shorter uptake time compared to FDG-PET.^25,55^ NaF is also less susceptible to spill-over artifact, such as from the myocardium, which can limit FDG-based PET imaging of the arteries.^67^ However, the cost, radiation exposure, and uptake and scanning time mean the role of PET imaging in routine clinical assessment of atherosclerotic disease is currently limited. There remain technical issues with PET imaging in atherosclerosis, mainly related to partial volume effects due to low spatial resolution and the small plaque volumes encountered. In addition, specific gating techniques are generally required to correct for cardiac motion over the image acquisition time, and there has been interest in different methodological approaches to correct for these issues.^68,69^

Microcalcification is known to confer an increased risk of plaque rupture through enzymatic^18^ and mechanical^17^ destabilization of the plaque surface. Therefore, targeting this process may reduce the risk of early recurrence following symptomatic atherosclerotic disease. NaF-PET can be utilized to assess responses to clinical interventions, given its accuracy and sensitivity in determining differences in the presence of microcalcification in vivo.^70^ Additionally, the process by which microcalcification evolves into macrocalcification, which is thought to be protective for the atherosclerotic plaque, is poorly defined and understood.^71^ Temporal evaluation of the microcalcification-macrocalcification process through NaF-PET/CT could shed more light on the factors which confer a greater or lesser degree of risk with calcification in atherosclerosis. In addition, NaF imaging could potentially have a role in risk stratification in patients where there is uncertainty about the risk of recurrent stroke or the need for surgical intervention, which is particularly relevant for those with moderate 50–69% stenoses and those where frailty or comorbidity may lead to a preference to avoid surgical intervention. In addition, there is interest in the use of NaF-PET imaging to identify study participants at higher risk of future clinical disease,^72^ or to provide surrogate endpoints in interventional trials.^73^

## Conclusion

Our findings support vascular NaF-PET imaging as a reliable method for the assessment of atherosclerotic disease that is able to help differentiate between culprit and nonculprit plaques within an individual but is unable to reliably differentiate between symptomatic and asymptomatic plaques in a population that includes unmatched asymptomatic individuals. The majority of data involve analysis of the carotid or coronary circulation, but NaF-PET imaging is also a viable imaging technique in other arterial territories. There is a potential future role of NaF-PET in clinical atherosclerosis imaging and providing surrogate markers of impact in interventional trials, but harmonization of imaging protocols including outcome measures, injected doses, and uptake times is required to ensure comparability between studies.

## Supplemental Material

sj-pdf-1-vmj-10.1177_1358863X241287692 – Supplemental material for 18F-NaF uptake on vascular PET imaging in symptomatic versus asymptomatic atherosclerotic disease: A meta-analysis Supplemental material, sj-pdf-1-vmj-10.1177_1358863X241287692 for 18F-NaF uptake on vascular PET imaging in symptomatic versus asymptomatic atherosclerotic disease: A meta-analysis by Shiv Bhakta, Mohammed M Chowdhury, Jason M Tarkin, James HF Rudd, Elizabeth A Warburton and Nicholas R Evans in Vascular Medicine
