# Supplementary material for: 18F-NaF uptake on vascular PET imaging in symptomatic versus asymptomatic atherosclerotic disease: A meta-analysis
Source: Vasc Med. 2024 Oct 16;30(1):10–9. doi: 10.1177/1358863X241287692 (PMC11804149; doi:10.1177/1358863X241287692)
Supplement: sj-pdf-1-vmj-10.1177_1358863X241287692 – Supplemental material for 18F-NaF uptake on vascular PET imaging in symptomatic versus asymptomatic atherosclerotic disease: A meta-analysis [file sj-pdf-1-vmj-10.1177_1358863X241287692.pdf]

Supplementary Table S1 Included study characteristics for studies comparing symptomatic to asymptomatic disease between arteries in the same participant

Author and Year	Article Type	Arterial Territory and Type of Symptomatic Disease	How was the symptomatic disease diagnosed?	Enrolled population demographics - number of participants, mean age and sex	Types of imaging used	Symptomatic patients completing 18F-NaF PET imaging (n), number of symptomatic plaques	Time from onset of symptoms to scan	Comparator population, number of comparator data points (n)	Dose of NaF injected, Uptake time	Imaging protocol	Endpoint measure	Blood Pool Measurement	Findings
Dweck[42], 2013	Abstract	Coronary, myocardial infarction	Not listed	n = 15, age/sex data not listed	FDG and NaF PET-CT	15, 15	Not listed	Maximum uptake elsewhere in the coronary arteries, 15	Not listed	Not listed	TBR (no further description)	Not listed	Increased uptake noted at the culprit lesion compared with the maximum uptake elsewhere in the coronary arteries (TBR median 1.56 [IQR 1.49-1.82] vs 1.23 [1.15-1.48], p=0.02)
Joshi[74], 2014	Paper	Coronary, ST-elevation myocardial infarction or non-ST-elevation myocardial infarction	Clinical guideline criteria	n = 40, age 62 ± 8 years, 93% male	FDG and NaF PET-CT, CT coronary angiogram, coronary angiogram	40, 40	Median 8 [3-10] days	Maximum signal value of the non-culprit vessels, 40	123 ± 5 MBq, 60 ± 9 minutes	Electrocardiograph-gated PET images were reconstructed in diastole	TBRmax - ROIs drawn around all major (diameter >2 mm) epicardial vessels on 3 mm axial slices just beyond the discernible adventitial border; 18F-NaF uptake in the culprit plaque was compared	corrected for blood pool activity in the superior vena cava	18F-NaF activity in the culprit plaque was 34% higher than the maximum activity recorded anywhere else in the coronary vasculature (maximum TBR 1.66 [1.40-2.25] vs 1.24 [1.06-1.38], p<0.0001)

											with the highest value in any of the non-culprit vessels		
Irkle[25], 2015	Paper	Carotid, stroke or transient ischemic attack	Not listed	n = 7, age 68.9 ± 13.5 years, 57.1% male	NaF PET-CT	5, 5	16.4 ± 8.0 days	PET-positive regions in asymptomatic participants, 2	Not listed	PET scanning performed for 75 minutes following tracer injection	"Mean of Max" TBR	Not listed	Individual participant data reported, and converted to median/interquartile range: symptomatic disease 2.48 [1.80-3.33] vs asymptomatic disease 2.41 [2.32-2.49], no p value.
Quirce[75], 2016	Short Report	Carotid, stroke or transient ischemic attack	Not listed	n = 9, age range 50-83 years, 88.9% male	FDG + NaF PET-CT	9, 9	Within 10 days	Contralateral artery, 9	370 MBq, 180 minutes	beds including the head and cervical region were acquired at 5 min per bed. Image data were reconstructed, and attenuation correction was done after applying iterative reconstruction methods with 2 iterations and 16 subsets.	TBRmax using SUVmax in the plaque	SUVmax in the lumen of the superior vena cava	Mean TBR was 2.12 ± 0.44 in the symptomatic plaques and 1.85 ± 0.46 in the asymptomatic (P = 0.22)
Vesey[76], 2017	Paper	Carotid, stroke or transient ischemic attack	Not listed	n = 26, demographic data listed separately: symptomatic carotid disease: n = 18, age 71.7 ± 12.3 years, 66.7% male;	FDG + NaF PET-CT, CT angiogram	14, 14	Not listed	Contralateral artery, 14	244.5 ± 12.66 MBq, 64.6 ± 5.6 minutes	PET acquisition covering 2 bed positions, 15 minutes per bed. PET data reconstructed with ordered subset expectation maximization+ point spread function	Three ROIs drawn on adjacent 3-mm axial slices. If plaque present, ROIs centered on area of highest	average of 5 ROIs within the lumen of the superior vena cava	Culprit 2.75 [2.39-3.21] vs contralateral 2.42 [2.02-2.82]; p=0.014 for log10 transformed distribution of original data

				asymptomatic carotid disease: n = 8, age 66.1 ± 12.5 years, 50% male						modeling+time-of-flight; 2 iterations and 21 subsets; matrix size 200x200; 5 mm full-width half-maximum Gaussian smoothing.	uptake. If no plaque present, uptake in the proximal 1 cm of internal carotid artery, just distal to the bifurcation was quantified.		
Andrews[43], 2018	Abstract	Coronary, myocardial infarction	Clinical + imaging	n = 13, age/sex data not listed	NaF PET-MRI, MR angiogram	13, 13	Not listed	Vessel proximal to culprit plaque, 13	Not listed	PET data acquired in list mode, Dixon attenuation correction technique	TBRmax - focal 18F-NaF uptake in culprit vessel	Not listed	13/13 patients with MI had focal 18F-NaF uptake in the culprit vessel with a TBR max greater than the proximal referent vessel (TBRmax 1.64±0.47 vs 1.16±0.26, p=0.004)
Marchesseau[77], 2018	Paper	Coronary, ST-elevation myocardial infarction	Clinical guideline criteria	n = 10, age 48 ± 7 years, 90% male	NaF PET-MRI + PET-CT, cardiac MRI, CT coronary artery calcium score scan	8, 8	Median 13.5 (range 9-24) days	Non-culprit plaques, 25	2.95 ± 0.21 mCi, 108 ± 21 minutes	10 minute acquisition, one bed position centered over the heart; reconstructed using an iterative reconstruction (3 iterations, 21 subsets, 2 mm pixel size, 2 mm FWHM Gaussian filter), with attenuation and scatter corrections included as well as Time of Flight and Point	TBRmax - ratio of the calcium region maximum SUV over the corresponding vessel (excluding the calcium ROIs) mean SUV	Mean SUV of the corresponding vessel minus the previously selected ROI	TBR of the culprit lesions was significantly higher than the TBR for non-culprit lesions with an average value of 2.11 for culprit (±0.42) and 1.36 (±0.30) for non-culprit (P value <0.001)

										Spread Function correction			
Sood[44], 2018	Abstract	Coronary, myocardial infarction	Clinical guideline criteria	n = 24, age/sex data not listed	NaF PET-CT, CT coronary angiogram	24, 24	Not listed	Non-culprit vessels, 72	3-5 mCi, 45 minutes	acquired in list mode	TBRmax from SUVmax of vessels	Not listed	The difference in median TBR of culprit vessels (1.46, IQR: 1.29-1.73) and non-culprit vessels (0.99, IQR: 0.80-1.02) was statistically significant (p-value <0.0001)
Zhang[78], 2018	Paper	Carotid, stroke, TIA, amaurosis fugax	Not listed	n = 8, age 60 ± 7.75 years, 87.5% male	NaF PET-CT	8, 8	Not listed	Contralateral artery, 8	4.44 MBq/kg, 60 minutes	2 bed positions centered over the carotid artery bifurcation, 3 minute 3D acquisition per bed	TBRmax - ROIs placed on hotspots around carotid bifurcation	3 ROIs in venous structures on the ipsilateral side	TBR_{max} of the operated side 2.415 ± 0.435 vs 2.389 ± 0.534 on the non-operated side
Chowdhury[79], 2020	Paper	Superficial femoral artery, peripheral vascular disease (intermittent claudication or critical limb ischemia)	Clinical + imaging	n = 50, median age 70 [65-78] years, 66% male	FDG and NaF PET-CT, CT angiogram	40, 40	Not applicable - participants included with intermittent claudication	Contralateral artery, 40	Mean 401.4 MBq, 60 minutes	PET data acquired from iliac crest to tibial plateau, 4 minute acquisition per bed position	TBRmax - ROIs placed on adjacent axial images, SUVmax per slice recorded	5 ROIs drawn in the center of the common femoral vein	Median baseline 18F-NaF TBRmax was 1.78 (IQR: 1.62 to 2.50); 1.54 (IQR: 1.36 to 1.72) in the contralateral (untreated, asymptomatic) limb, no p value reported
Evans[57], 2020	Paper	Carotid, stroke	Clinical + imaging	n = 31, age 74.8 ± 9.7 years, 69.2% male	FDG and NaF PET-CT, CT angiogram	26, 26	8.5 ± 4.2 days to initial scan, 53.8% had NaF PET first, median 1 [IQR 1.75] days between	Contralateral artery, 26	125 MBq, 60 minutes	ROIs drawn along the common carotid and internal carotid artery to encompass the region 0.9 cm proximal and 3 cm distal to the carotid bifurcation	MDS TBRmax: MDS uses most diseased 9 mm, based on tracer uptake 3 contiguous axial slices where the central ROI	mid-luminal ROIs in the jugular vein over 5 contiguous 3 mm slices	NaF uptake was higher in culprit plaques than nonculprit plaques when considering MDS TBRmax - mean 2.85 +/- 1.15 vs 2.34 +/- 0.64, p<0.01

							NaF and FDG scans				has the highest uptake within the artery		
Kaczynski[47], 2022	Paper	Carotid, stroke, transient ischemic attack or amaurosis fugax	Clinical + imaging	n = 110, age 68 ± 10 years, 64% male	NaF PET-MRI, MR angiogram	110, 110	Not listed	Contralateral artery, 110	125 MBq, 60 minutes	PET data obtained using list-mode acquisitions with two 15-min beds positions covering the carotid arteries (aortic arch to skull base). Images reconstructed with corrections applied for attenuation, dead time, scatter and random coincidences (matrix size 256x256, ordered subset expectation maximization reconstruction with point spread function modelling, 3 iterations, 21 subsets, 2mm Gaussian filtration)	TBRmax - uptake measured in a spherical VOI centered on the most severe stenosis	averaged from 3 ROIs within the brachiocephalic or internal jugular veins	Culprit vessels had a higher 18F-NaF uptake than non-culprit vessels (TBRmax 1.38 [1.12, 1.82] versus 1.26 [0.99, 1.66], p=.04)

Supplementary Table S2 Included study characteristics for studies comparing symptomatic to asymptomatic disease within and between participants

Author, Year	Article Type	Arterial Territory, Type of Symptomatic Disease	How symptomatic disease was diagnosed	Participant demographics - number of participants, mean age and sex	Types of imaging used	Symptomatic patients completing 18F-NaF PET imaging (n), number of symptomatic plaques	Time from onset of symptoms to scan	Comparator population, number of comparator data points (n)	Dose of NaF injected, Uptake time	PET Imaging protocol	Endpoint measure	Blood Pool Measurement	Findings
Joshi[74], 2014	Paper	Coronary, ST-elevation myocardial infarction or non-ST-elevation myocardial infarction	Clinical guideline criteria	n = 40, age 62 ± 8 years, 93% male	FDG and NaF PET-CT, CT coronary angiogram, coronary angiogram	40, 40	Median 8 [3-10] days	PET-positive plaques in participants with stable angina, 15; PET-negative plaques in participants with stable angina, 24	123 ± 5 MBq, 60 ± 9 minutes	Electrocardiograph-gated PET images were reconstructed in diastole	TBRmax - ROIs drawn around all major (diameter >2 mm) epicardial vessels on 3 mm axial slices just beyond the discernible adventitial border; 18F-NaF uptake in the culprit plaque was compared with the highest value in any of the non-culprit	corrected for blood pool activity in the superior vena cava	Culprit plaque maximum TBR 1.66 [1.40-2.25] vs 1.90 [1.61-2.17] in PET-positive plaques in stable angina participants, 1.02 [0.82-1.17] in PET-negative plaques in stable angina participants

											vessels		
Irkle[25], 2015	Paper	Carotid, stroke or transient ischemic attack	Not listed	n = 7, age 68.9 ± 13.5 years, 57.1% male	NaF PET-CT	5, 5	16.4 ± 8.0 days	PET-negative regions in symptomatic participants, 5, and PET-positive and negative regions in asymptomatic participants, 4	Not listed	PET scanning performed for 75 minutes following tracer injection	"Mean of Max" TBR	Not listed	Individual participant data reported, and converted to median/interquartile range: symptomatic disease 2.48 [1.80-3.33] vs asymptomatic disease 0.98 [0.95-1.27], no p value.
Cocker[80], 2017	Short Report	Carotid, stroke or transient ischemic attack	Not listed	n = 11, age 69 ± 5 years, 72.7% male	NaF PET-CT, CT angiogram	9, 9	Not listed	Contralateral artery or asymptomatic carotid arteries, 11	3 MBq/kg, 60 minutes	PET/CT imaging was performed, followed by CT angiography	TBRmax - maximum 18F-NaF activity for each plaque	Normalized to mean SUV in internal jugular vein	Plaque associated with symptoms had evidence for greater 18F-NaF uptake than plaque not associated with symptoms (TBRmax: 3.75 ± 1.10 vs. 2.79 ± 0.60; p = 0.04)
Vesey[76], 2017	Paper	Carotid, stroke or transient ischemic attack	Not listed	n = 26, demographic data listed separately: symptomatic carotid disease: n = 18, age 71.7 ± 12.3 years, 66.7% male; asymptomatic carotid disease: n = 8, age 66.1 ± 12.5 years, 50% male	FDG + NaF PET-CT, CT angiogram	14, 14	Not listed	Unpaired asymptomatic control participant, 12	244.5 ± 12.66 MBq, 64.6 ± 5.6 minutes	PET acquisition covering 2 bed positions, 15 minutes per bed. PET data reconstructed with ordered subset expectation maximization+point spread function modeling+time-of-flight; 2 iterations and 21 subsets; matrix size 200x200; 5 mm full-width half-maximum Gaussian smoothing.	Three ROIs drawn on adjacent 3-mm axial slices. If plaque present, ROIs centered on area of highest uptake. If no plaque present, uptake in the proximal 1 cm of internal	average of 5 ROIs within the lumen of the superior vena cava	Culprit 2.75 [2.39-3.21] vs control 2.44 [1.715-2.48]; p=0.016 for log10 transformed distribution of original data

											carotid artery, just distal to the bifurcation was quantified.		
Sood[44], 2018	Abstract	Coronary, myocardial infarction	Clinical guideline criteria	n = 24, age/sex data not listed	NaF PET-CT, CT coronary angiogram	24, 24	Not listed	High risk plaques in participants with stable angina, 29 Low risk plaques in participants with stable angina, 39	3-5 mCi, 45 minutes	acquired in list mode	TBRmax from SUVmax of vessels	Not listed	Median TBR of culprit vessels (1.46, IQR: 1.29-1.73), 1.25 [0.77-3.57] for high risk plaques in stable angina, and 1.08 [0.53-2.26] in low risk plaques in stable angina
Hop[81], 2019	Paper	Carotid, stroke	Clinical + imaging	n = 23, median age 72 [61-75] years, 85% male	NaF microPET, microCT	17, 17	21 ± 14 days	Endarterectomy specimens from non-culprit plaques, 6	49.4 ± 7.2 MBq (incubated), 60 minutes	MicroPET emission scan for 30 minutes, reconstructed using OSEM2D (4 iterations and 16 subsets), after being normalized and corrected for attenuation and decay of radioactivity.	%Inc/g – Percentage uptake of total incubation dose per gram of tissue (%Inc/g).	N/A	Average 18F-NaF uptake was similar in culprit and non-culprit carotid plaques (median 2.32 %Inc/g [IQR 1.98 to 2.81] vs. median 2.35 %Inc/g [IQR 1.77 to 3.00], p = 0.916)
Kim[82], 2019	Paper	Carotid, stroke or transient ischemic attack	Clinical + imaging	n = 18, demographic data listed separately: symptomatic group: n = 10, age 72.9 ± 8.2 years, 50% male; asymptomatic group: n = 8, age 77.1 ± 10.9 years, 50%	FDG and NaF PET-CT, CT angiogram	10, 10	Median 17 (range 3-37) days	Contralateral artery or asymptomatic carotid arteries in those with an alternative cause of stroke, 26	259-370 MBq, 60 minutes	PET images acquired at 5 min/bed for the head and 1 min/bed from the skull base to the proximal thigh	NaF TBRmax at the largest atheroma segment	SUV of aortic blood	NaF TBRmax at the largest atheroma segment in symptomatic vs asymptomatic plaques: 1.53 ± 0.54 vs 1.39 ± 0.45, p = 0.37

				male									
Mechtouff [83], 2022	Paper	Carotid, stroke or transient ischemic attack	Not listed	n = 12, age 68 ± 10 years, 75% male	NaF PET-MRI, MR angiogram	6, 6	9.2 ± 6.2 days	Contralateral artery and carotid arteries of asymptomatic control participants, 18	3 MBq/kg, 60 minutes	15 minute PET acquisition	TBRmax measured using 3 ROIs centered on the area of highest uptake in the plaque	mean of five ROIs in the mid lumen of the superior vena cava	18F-NaF uptake was higher in culprit plaques compared to nonculprit plaques (median TBR 2.6 [2.2-2.8] vs 1.7 [1.3-2.2]; P = 0.03)

Embase <1996 to 2023 Week 30>
Ovid MEDLINE(R) and Epub Ahead of Print, In-Process, In-Data-Review & Other Non-Indexed Citations, Daily and Versions <1946 to August 02, 2023>

1	(18F* or NaF or $F-NaF*).ti,ab.
2	(PET or positron*).ti,ab.
3	(athero* or acute or microcalcif* or plaque).ti,ab.
4	(culprit or symptom* or active).ti,ab.
5	1 and 2 and 3 and 4
6	(editorial* or case report* or review* or meta-analysis).pt.
7	5 not 6

Supplementary Figure S1a: Search strategy for the Ovid Embase and MEDLINE databases

−		Title Abstract Keyword	18F* or NaF or $NaF*
−	AND	Title Abstract Keyword	PET or positron*
−	AND	Title Abstract Keyword	athero* or acute or microcalcif* or plaque
−	AND	Title Abstract Keyword	culprit or symptom* or active

(Word variations have been searched)

Supplementary Figure S1b: Search strategy for the Cochrane Library database

TITLE-ABS-KEY (18f* OR naf OR $f-naf*) AND TITLE-ABS-KEY (pet OR positron*) AND TITLE-ABS-KEY (athero* OR acute OR microcalcif* OR plaque) AND TITLE-ABS-KEY (culprit OR symptom* OR active) AND NOT DOCTYPE (editorial* OR case AND report* OR review* OR meta-analysis)

Supplementary Figure S1c: Search strategy for the PubMed database

Search: **(18F or NaF or *F-NaF*) and (PET or positron*) and (athero* or acute or microcalcif* or plaque) and (culprit or symptom* or active)**
("18F"[All Fields] OR "NaF"[All Fields] OR "f naf"[All Fields]) AND ("PET"[All Fields] OR "positron*"[All Fields]) AND ("athero*"[All Fields] OR ("acute"[All Fields] OR "acutely"[All Fields] OR "acutes"[All Fields]) OR "microcalcif*"[All Fields] OR ("plaque s"[All Fields] OR "plaque, amyloid"[MeSH Terms] OR ("plaque"[All Fields] AND "amyloid"[All Fields]) OR "amyloid plaque"[All Fields] OR "plaque"[All Fields] OR "dental plaque"[MeSH Terms] OR ("dental"[All Fields] AND "plaque"[All Fields]) OR "dental plaque"[All Fields] OR "plaques"[All Fields])) AND ("culprit"[All Fields] OR "culprits"[All Fields] OR "symptom*"[All Fields] OR ("activable"[All Fields] OR "activate"[All Fields] OR "activated"[All Fields] OR "activates"[All Fields] OR "activating"[All Fields] OR "activation"[All Fields] OR "activations"[All Fields] OR "activator"[All Fields] OR "activator s"[All Fields] OR "activators"[All Fields] OR "active"[All Fields] OR "actived"[All Fields] OR "actively"[All Fields] OR "actives"[All Fields] OR "activities"[All Fields] OR "activity s"[All Fields] OR "activitys"[All Fields] OR "motor activity"[MeSH Terms] OR ("motor"[All Fields] AND "activity"[All Fields]) OR "motor activity"[All Fields] OR "activity"[All Fields]))
Translations
acute: "acute"[All Fields] OR "acutely"[All Fields] OR "acutes"[All Fields]
plaque: "plaque's"[All Fields] OR "plaque, amyloid"[MeSH Terms] OR ("plaque"[All Fields] AND "amyloid"[All Fields]) OR "amyloid plaque"[All Fields] OR "plaque"[All Fields] OR "dental plaque"[MeSH Terms] OR ("dental"[All Fields] AND "plaque"[All Fields]) OR "dental plaque"[All Fields] OR "plaques"[All Fields]
culprit: "culprit"[All Fields] OR "culprits"[All Fields]
active: "activable"[All Fields] OR "activate"[All Fields] OR "activated"[All Fields] OR "activates"[All Fields] OR "activating"[All Fields] OR "activation"[All Fields] OR "activations"[All Fields] OR "activator"[All Fields] OR "activator's"[All Fields] OR "activators"[All Fields] OR "active"[All Fields] OR "actived"[All Fields] OR "actively"[All Fields] OR "actives"[All Fields] OR "activities"[All Fields] OR "activity's"[All Fields] OR "activitys"[All Fields] OR "motor activity"[MeSH Terms] OR ("motor"[All Fields] AND "activity"[All Fields]) OR "motor activity"[All Fields] OR "activity"[All Fields]

Supplementary Figure S1d: Search strategy for the Scopus database

1: TI=(18F* or NaF or $F-NaF*) Results: 45346

2: AB=(18F* or NaF or NaF*) Results: 69564

3: #1 OR #2 Results: 108323

4: TI=(PET or positron*) Results: 161782

5: AB=(PET or positron*) Results: 208154

6: #4 OR #5 Results: 286689

7: TI=(athero* or acute or microcalcif* or plaque) Results: 840904

8: AB=(athero* or acute or microcalcif* or plaque) Results: 1299541

9: #7 OR #8 Results: 1769725

10: TI=(culprit or symptom* or active) Results: 614807

11: AB=(culprit or symptom* or active) Results: 3119599

12: #10 OR #11 Results: 3401093

13: #3 AND #6 AND #9 AND #12 Results: 292

Supplementary Figure S1e: Search strategy for the Web of Science Core Collection database

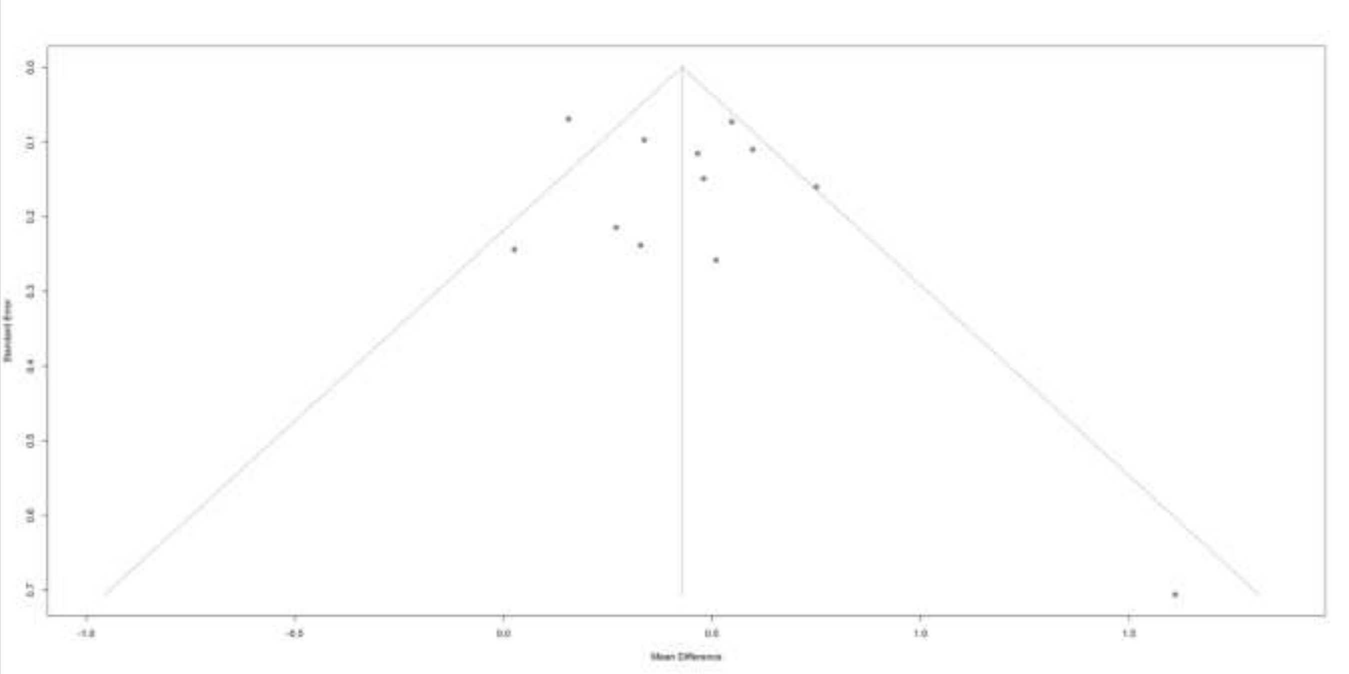

Supplementary Figure S2: Funnel plot of studies included in Supplementary Table 1[25, 41, 52, 69-77]

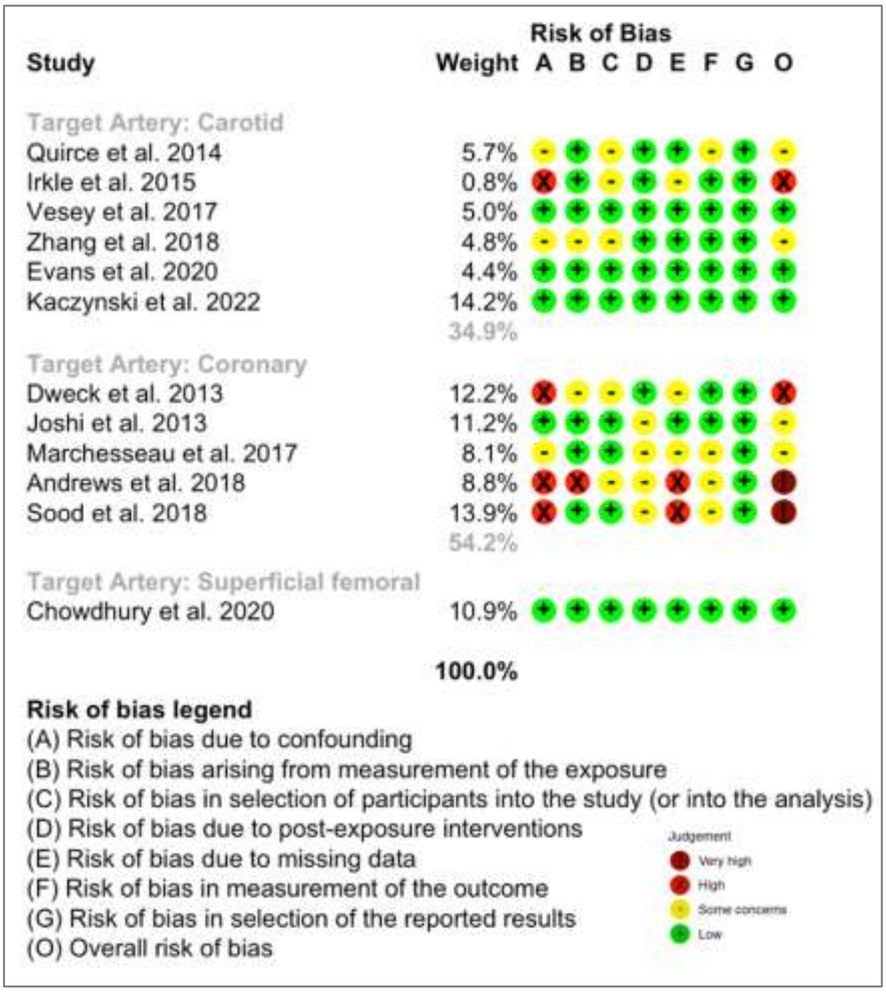

Supplementary Figure S3: Risk of Bias assessment of included studies[25, 41, 52, 69-77] summarizing data comparing symptomatic and asymptomatic atherosclerotic disease within individuals, using the ROBINS-E tool.

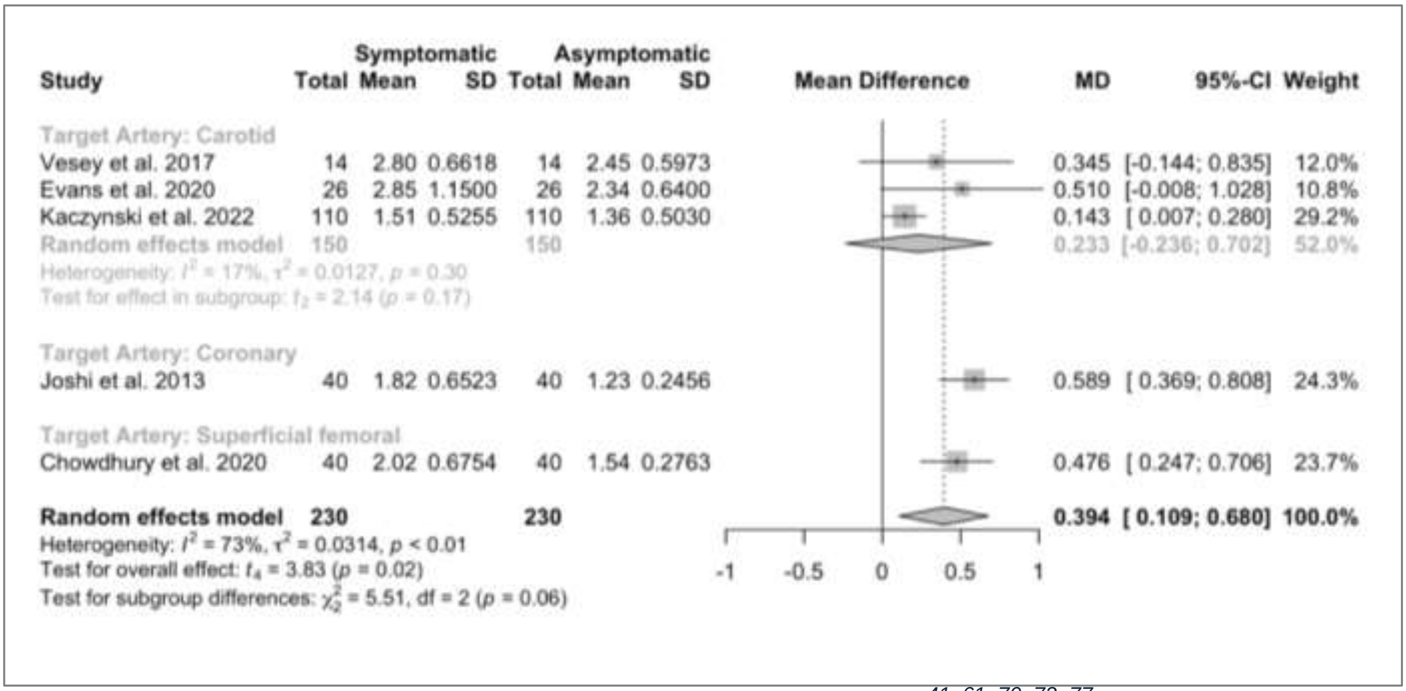

Supplementary Figure S4: Sensitivity analysis of studies included[41, 61, 70, 72, 77] in the intra-individual analyses judged to be at low risk of bias, or some concerns in one domain, due to the need for percutaneous coronary intervention in the myocardial infarction group

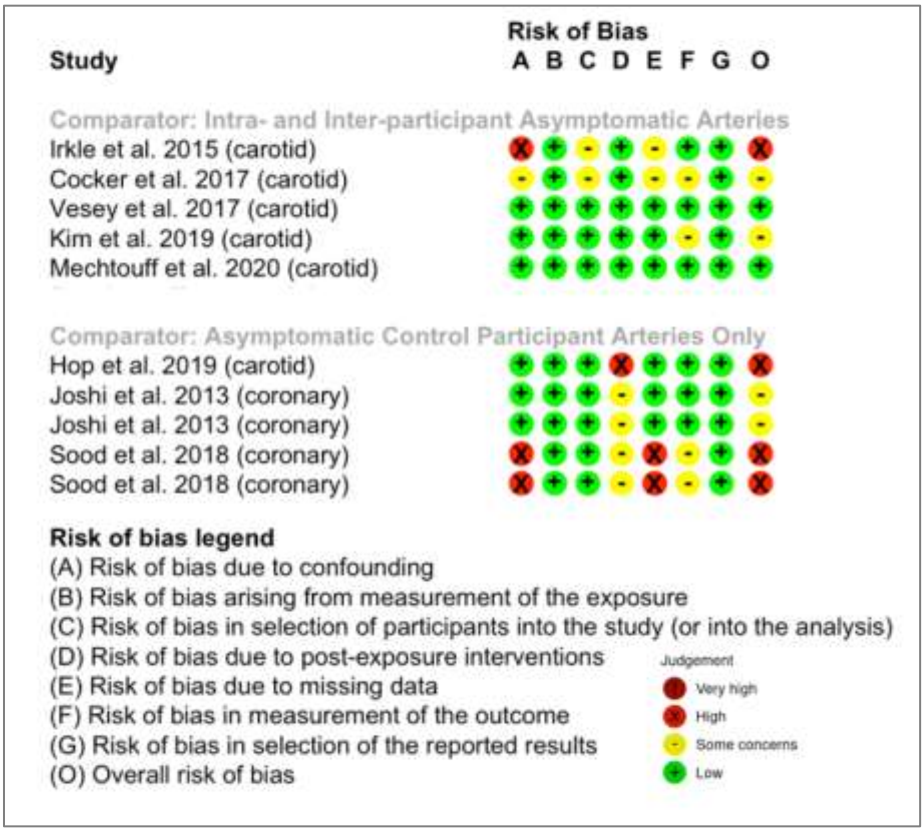

Supplementary Figure S5: Risk of Bias assessment of included studies[25, 70, 72, 75, 78-81] summarizing data comparing symptomatic and asymptomatic atherosclerotic disease between individuals, using the ROBINS-E tool.
